# FTIR Spectroscopic and Molecular Analysis during Differentiation of Pluripotent Stem Cells to Pancreatic Cells

**DOI:** 10.1155/2016/6709714

**Published:** 2016-08-29

**Authors:** Gustavo Jesus Vazquez-Zapien, Monica Maribel Mata-Miranda, Virginia Sanchez-Monroy, Raul Jacobo Delgado-Macuil, David Guillermo Perez-Ishiwara, Marlon Rojas-Lopez

**Affiliations:** ^1^CIBA-Tlaxcala, Instituto Politécnico Nacional, 90700 Tepetitla, TLAX, Mexico; ^2^Laboratorio de Embriología, Escuela Médico Militar, Universidad del Ejército y Fuerza Aérea, 11200 Ciudad de México, Mexico; ^3^Laboratorio de Biología Celular y Tisular, Escuela Médico Militar, Universidad del Ejército y Fuerza Aérea, 11200 Ciudad de México, Mexico; ^4^Laboratorio Multidisciplinario de Investigación, Escuela Militar de Graduados de Sanidad, Universidad del Ejército y Fuerza Aérea, 11200 Ciudad de México, Mexico; ^5^Escuela Nacional de Medicina y Homeopatía, Instituto Politécnico Nacional, 07320 Ciudad de México, Mexico

## Abstract

Some of the greatest challenges in stem cells (SCs) biology and regenerative medicine are differentiation control of SCs and ensuring the purity of differentiated cells. In this work, we differentiated mouse pluripotent stem cells (mPSCs) toward pancreatic cells characterizing this differentiation process by molecular and spectroscopic technics. Both mPSCs and Differentiated Pancreatic Cells (DPCs) were subjected to a genetic, phenotypic, and biochemical analysis by real-time quantitative PCR (RT-qPCR), immunocytochemistry, and Fourier Transform Infrared (FTIR) spectroscopy. Cultured mPCSs expressed pluripotent genes and proteins (*Nanog* and* SOX2*). DPCs expressed endodermal genes (*SOX17* and* Pdx1*) at day 11, an inductor gene of embryonic pancreas development (*Pdx1*) at day 17 and pancreas genes and proteins (*Insulin* and* Glucagon*) at day 21 of differentiation. Likewise, FTIR spectra of mPSCs and DPCs at different maturation stages (11, 17, and 21 days) were obtained and showed absorption bands related with different types of biomolecules. These FTIR spectra exhibited significant spectral changes agreeing with the differentiation process, particularly in proteins and nucleic acids bands. In conclusion, the obtained DPCs passed through the chronological stages of embryonic pancreas development and FTIR spectra provide a new biophysical parameter based on molecular markers indicating the differentiation process of mPSCs to specialized cells.

## 1. Introduction

Currently, world healthcare system is being overwhelmed by the increase in average life expectancy worldwide, leading to increase in the prevalence of several chronic degenerative diseases as well as their complications. Unfortunately, actual treatment options for most of these diseases do not slow any further deterioration or restore the function; rather they only treat the symptoms or partially replace the function, accumulating comorbidities and the consequent health deterioration.

Current stem cells (SCs) researches have opened new possibilities for the treatment of chronic diseases, and cell therapy now stands at the front-line of regenerative medicine and tissue engineering [1]. SCs can be used to improve healthcare, augmenting the regenerative potential of the body or for the development of new therapies [2].

An essential property of SCs is their potentiality, which refers to their capability to differentiate into different cell lineages under certain culture conditions. According to their origin and differentiation potential, SCs are classified as totipotent, pluripotent, multipotent, and unipotent. Pluripotent stem cells (PSCs) commonly are obtained from the Inner Cell Mass (ICM) and can give arise to the three germ layers (ectoderm, mesoderm, and endoderm), differentiating or developing into more than 200 specialized cells [3].

Experimental studies in animals and some clinical trials employing SCs have shown the therapeutic potential of SCs and have brought hope to patients suffering devastating pathologies of different organs and systems as diabetes, renal failure, and ocular diseases, among others [4–6].

Diabetes is a chronic disease that represents a serious public health problem and occurs either when the pancreas does not produce enough insulin or when the body cannot effectively use the insulin it produces. In 2014, 9% of adults aged 18 years or older had diabetes, and in 2012 this condition represented the direct cause of 1.5 million deaths on the world [7]. Current diabetes treatments do not reverse the disease; besides, in many cases the therapeutic effects are insufficient for the metabolic control. Therefore, alternative sources for *β*-cell replacement are being studied, including embryonic stem cells (ESCs), induced pluripotent stem cells, and mesenchymal stem cells [8]. In this sense, several protocols that described the derivation of pancreatic cells from pluripotent stem cells (PSCs)* in vitro* are being carried out [9].

Two of the greatest challenges in SCs biology and regenerative medicine are differentiation control of SCs and ensuring the purity of differentiated cells [10]. Therefore, Differentiated Pancreatic Cells (DPCs) from SCs must be characterized before being implanted in animal models; thus, various specialized techniques as flow cytometry, immunocytochemistry, and real-time quantitative PCR (RT-qPCR) assays have been used in this field [11–13]. Nevertheless, although the aforementioned techniques are very efficient and require long time, specialized personnel, and sometimes a great amount of cells, for these reasons it is necessary to propose efficient, rapid, and noninvasive techniques that could help to facilitate the identification of cells or maturation stages along cell differentiation process.

Fourier Transform Infrared (FTIR) spectroscopy is a powerful technique used to obtain the molecular fingerprint in a sample, which absorbs the IR wave according to the structural and chemical bonds of molecules of biological samples, providing information about specific structure of biomolecules such as proteins, lipids, carbohydrates, and nucleic acids, giving rise to a series of identifiable functional group bands in the mid infrared electromagnetic region [14]; some authors have reported the use of FTIR spectroscopy for potential applications in biological systems, which include cytological, histological, and microbial studies, approaching to clinical diagnosis in combination with computational techniques. For this reason, biospectroscopy is becoming a common tool in the screening or diagnostic laboratory [15].

On the other hand, the use of FTIR spectroscopy in SCs researches is very wide; it has been used as a tool to detect changes in the macromolecular content of SCs and their subsequent differentiation across the absorption of electromagnetic radiation in the middle infrared range (from 4000 to 400 cm^−1^ wavenumbers) [16]. Diverse research groups have successfully characterized different SCs types and differentiated cells from ESCs; some others have elucidated biochemical differences between murine and human SCs [17]; the determination of chemical differences between cells from the same specie with different potentiality has also been shown [18]; and differences between spectra of mouse ESCs (mESCs) and human ESCs (hESCs) lines and their derived cell types have been detected after several days of differentiation [16, 19], among other applications.

The aim of this work was to induce the differentiation of mouse pluripotent stem cells (mPSCs) toward DPCs. Along differentiation, these cells were subjected to a genetic, phenotypic, and biochemical analysis by RT-qPCR, immunofluorescence, and infrared vibrational spectroscopy, emphasizing that until today there are not reported experimental works that combine the aforementioned techniques to characterize DPCs from mPSCs at specific stages of differentiation. Furthermore, it is worth to note that FTIR vibrational spectroscopy has not been used for monitoring this differentiation process.

## 2. Materials and Methods

### 2.1. Pluripotent Stem Cells Culture

Mouse pluripotent stem cells (ATCC; SCRC-1011) were seeded at a density of 50,000 cells/cm^2^ on monolayers of mitotically inactive Mouse Embryonic Fibroblasts (MEFs) feeder cells (STO: S, SIM; T, resistant to thioguanine; O, resistant to ouabain) in order to prevent differentiation. We used mouse ESC basal medium (ATCC; SCRR-2010), supplemented with 15% Fetal Bovine Serum (FBS) (ATCC; 30-2020), 0.1 mM 2-mercaptoethanol (Invitrogen; 21985023), and 1,000 U/mL mouse leukemia inhibitory factor (Chemicon; ESG1107). Before mPSCs were subjected to a differentiation protocol, these cells were separated from the monolayer of MEFs.

### 2.2. Differentiation of mPSCs into Differentiated Pancreatic Cells

To direct differentiation of mPSCs toward DPCs and promote the consecutive formation of Embryoid Bodies (EBs), mPSCs were seeded at a density of 50,000 cells/cm^2^; subsequently, these cells were subjected to differentiation using a multistep differentiation protocol of 21 days which is described as follows: 4 differentiation mediums were used at different times, the differentiation mediums 1 and 2 were used on days 0 and 2 of differentiation, respectively, containing Iscove's Modified Dulbecco's Medium (IMDM) (Sigma; 51471C) supplemented with 15% Fetal Calf Serum (FCS) (Promocell; C-37350), 50 *μ*g/mL Ascorbic Acid (AA) (Sigma; A5960), and Monothioglycerol (MTG) (Sigma; M4165) at a concentration of 6 × 10^3^ and 6 × 10^4^ M for differentiation mediums 1 and 2, respectively. The differentiation medium 3 was used from day 6 to day 13 of differentiation, and it consisted of Dulbecco's Modified Eagle Medium: Nutrient Mixture F12 (DMEM/F12) (Invitrogen, 11320-033) supplemented with 15% FBS and 10 ng/mL growth factor purified recombinant human fibroblasts (FGF2) (Sigma, F0291); finally, from day 13 until the end of the differentiation protocol (day 21), EBs were maintained on differentiation medium 4 containing differentiation medium 3 plus 10 mM nicotinamide (Sigma, N0636) 0.1 nM exendin-4 (American Peptide Company; 46-3-12) and 10 ng/mL recombinant human activin-B (Sigma; A1729).

All cell types were incubated at 37°C in a humidified incubator (5% CO_2_, 95% air), once mPSCs cultures reached 75% confluence, and DPCs fulfilled specific time points of differentiation (11, 17, and 21 days); each cell type was analyzed by triplicate through genetic, protein, and spectroscopic assays.

### 2.3. RT-qPCR Assays

Total RNA of mPSCs and DPCs at different maturation stages (11, 17, and 21 days of differentiation) was isolated using Trizol reagent (Invitrogen; 15596-018), as per the manufacturers' instructions (Invitrogen; 15596-018); thereafter cDNA synthesis was performed using the first strand cDNA synthesis kit (Invitrogen; 12328-040) following the manufacturer's instructions. RT-qPCR was conducted by using ABI PRISM 7000 Sequence Detection System (Applied Biosystems, USA). At each cycle, accumulation of PCR products was detected by monitoring the increase in fluorescence of the reporter SYBR Green PCR Master Mix (Applied Biosystems; 4309155). Straightaway after the amplification, dissociation curves were run and analyzed to ensure the specificity of the PCR product. The relative expression levels were calculated using the CT method, which uses the arithmetic formula 2^−ΔΔCT^. Relative RNA levels of all tested genes were normalized to peptidylprolyl isomerase A (*ppia*) housekeeping gene and were expressed as means ± standard deviation (SD).

Primers were designed using the Primer Express Software for real-time PCR ver 3.0 (Applied Biosystems) (Table 1).

### 2.4. Immunocytochemistry

To characterize phenotypically the mPSCs and DPCs on day 21 of differentiation, both cell lines were seeded in chamber slide (Sigma-Aldrich); after mPSCs reached 75% confluence and DPCs fulfilled 21 days on differentiation, both cell lines were fixed in 4% paraformaldehyde (Sigma; P6148) for 30 minutes; thereafter, samples were rinsed with phosphate buffer solution (PBS) twice. Afterward, fixed cells were permeabilized with 0.1% Triton X100 (Sigma; X100) in PBS at room temperature for 5 minutes, and after that samples were rinsed with PBS. Subsequently, cells were blocked using blocking protein (Dako; X0909) for 20 minutes to inhibit nonspecific staining. Immunocytochemistry staining was done using rabbit primary antibodies anti-Nanog (1 : 200, Abcam; ab80892), anti-SOX2 (1 : 250, Abcam; ab97959), anti-glucagon (1 : 50, Dako; A0565), and guinea pig primary antibody anti-insulin (1 : 20, Dako; A0564); PSCs antibodies were incubated overnight at 4°C and pancreas antibodies were incubated for 60 minutes at room temperature. Subsequently, samples were washed with PBS twice and the conjugated secondary antibodies, dylight 488 goat anti-rabbit (1 : 200, Abcam; AB96895), and alexa fluor 488 goat anti-guinea pig (1 : 200, Jackson; 706-546-148) were incubated for 45 minutes in darkness. Finally, samples were washed with PBS and coverslipped with 10% glycerol. Microscopic observations were performed in a fluorescence microscopy (Ti-U Eclipse, Nikon, Japan).

### 2.5. Fourier Transform Infrared Spectroscopy

FTIR spectral analysis of mPSCs and DPCs taken at different maturation stages (11, 17, and 21 days of differentiation) was conducted in the spectral range between 400 and 4000 cm^−1^ using a FTIR spectrometer Bruker Vertex 70 in the Attenuated Total Reflection (ATR) sampling mode. Three replicates of each differentiation stage (0, 11, 17, and 21 days) were prepared to be analyzed by FTIR spectroscopy, for which propose cells samples were collected and washed twice using PBS in order to eliminate medium contamination; thereafter samples were centrifuged at 1200 rpm for 3 minutes and the supernatant was removed. Finally, the cell suspension (about 3 *μ*L containing ~10^5^ cells) was deposited onto the surface of the ATR crystal and dried at room temperature for about 15 minutes to eliminate water excess. The infrared radiation propagates along the crystal to obtain the corresponding spectra which was the average of 120 data acquisitions.

### 2.6. Statistical Analysis

All data were performed in triplicate, and all experiments were repeated at least three times. Data were presented as mean ± SD and analyzed using one-way analysis of variance (ANOVA), followed by a Tukey's test to determine any significant differences. *P* values of less than 0.05 were considered statistically significant.

## 3. Results

### 3.1. Morphological Description

As mentioned in previous reports [3], microscopic observations on mPSCs in culture showed that these cells tend to grow in colonies, which is a morphological characteristic of the pluripotent state. Thereafter, mPSCs were subjected to an* in vitro* differentiation protocol of 21 days, observing the formation of EBs, that were growing according culture time.

### 3.2. Gene Expression

**Figure 1:**
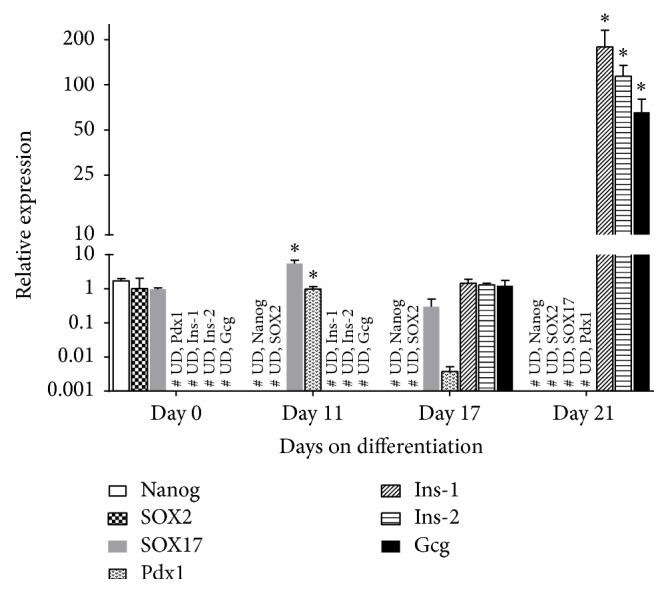
Mouse pluripotent stem cell cultures were assayed for relative expression levels of pluripotency (Nanog, SOX2), endodermal (SOX17, Pdx1), and pancreatic (Ins-1, Ins-2, and Gcg) genes at different points in time. RT-qPCR was performed in triplicate for each sample with bars representing means ± SD of 3 biological replicates. Expression levels were normalized against the housekeeping gene* ppia*. Asterisks (*∗*) denote statistical significant (*P* < 0.001) increases in pancreatic marker gene expression at specific time points of cultures as compared to nondifferentiated mouse pluripotent stem cells (day 0 of differentiation), # UD represents the undetected genes.

The expression of pluripotency (*Nanog* and* SOX2*), genes involved in endodermal germ line (*SOX17* and* Pdx1*), an inductor gene of embryonic pancreas development (*Pdx1*), and pancreas genes (*Insulin-1*,* Insulin-2,* and* Glucagon*) were studied in both mPSCs and DPCs. Changes in relative expression by RT-qPCR of the aforementioned genes at specific maturation stages (0, 11, 17, and 21 days of differentiation) are summarized in Figure 1.

The results showed that mPSCs (day 0 of differentiation) expressed* Nanog* and* SOX2* genes, but both genes were downregulated, and their expression was undetectable at day 11 of differentiation. Regarding* SOX17, *this gene was expressed at day 0 (0.96-fold), increasing thereafter its expression, exhibiting at day 11 its highest level of expression, 5.47-fold higher than in mPSCs, and at day 17 of differentiation, the expression decreased to 0.29-fold.* Pdx1* expression was detectable until day 11 of differentiation (0.99-fold), and at day 17 the expression was almost undetectable (0.003-fold). Concerning pancreas genes,* Insulin-1* (*Ins-1*) and* Insulin-2* (*Ins-2*) were expressed until day 17 of differentiation, 1.44- and 1.29-fold, respectively; thereafter their expression significantly increased up to 178.41- and 114.19-fold, respectively, at the end of the differentiation protocol, and finally,* Glucagon* (*Gcg*) was slightly expressed until day 17 of differentiation (1.21-fold), increasing thereafter to 46.35-fold at day 21 of differentiation.

### 3.3. Immunofluorescence Staining

**Figure 2:**
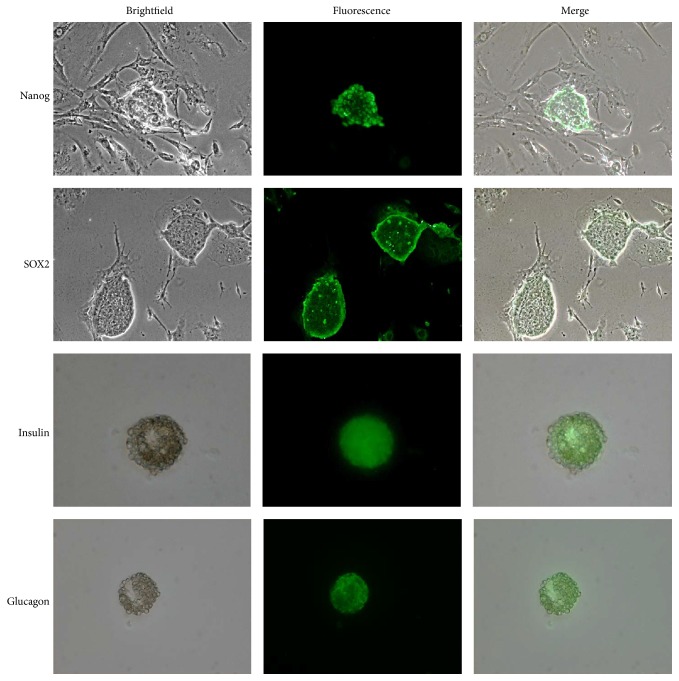
Representative images of immunofluorescence on mouse pluripotent stem cells (mPSCs) and Differentiated Pancreatic Cells (DPCs). The pluripotency markers Nanog and SOX2 were immunodetected on mPSCs cultures, evidencing the pluripotency state. The immunoexpression of pancreas proteins (Insulin and Glucagon) in the Embryoid Bodies (EBs) corroborated the obtention of pancreatic cells (*N* = 3 with 3 biological replicates, 100x, Insulin 200x).

In agreement with gene expression results, the analysis of protein expression in mPSCs by immunofluorescence confirmed the expression of Nanog and SOX2 proteins (pluripotent markers). After confirming the pluripotent state of mPSCs, these cells underwent differentiation, forming EBs, which immunoexpressed insulin and glucagon proteins at day 21 of differentiation, supporting the obtention of DPCs (Figure 2).

### 3.4. FTIR Analysis

**Figure 3:**
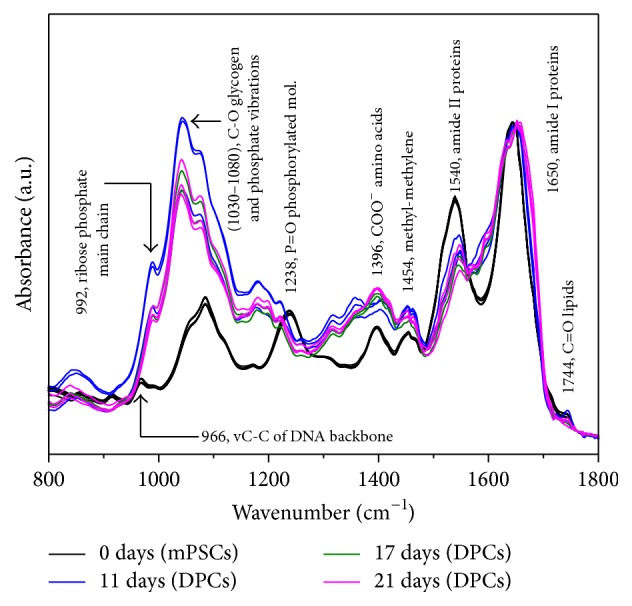
FTIR spectra of mouse pluripotent stem cells (mPSCs) and Differentiated Pancreatic Cells (DPCs) at different maturation stages (11, 17, and 21 days) depicted in the fingerprint region (*N* = 3).

FTIR spectra of mPSCs and DPCs at different maturation stages (11, 17, and 21 days) are shown in Figure 3. We obtained absorption bands related with different types of biomolecules including lipids, proteins, carbohydrates, and nucleic acids, which are normally present in cells. Firstly, a weak band at 1744 cm^−1^ which arises from the C=O stretching mode of lipids is observed. Two intense bands at 1650 and 1540 cm^−1^ are associated with the amide I (C=O stretching) and amide II (N-H bending) functional groups of proteins, respectively. Another band at 1454 cm^−1^ is commonly related with methyl methylene groups from lipids and proteins, whereas the band at 1396 cm^−1^ arises from the COO^−^ stretching vibrations of amino acid side chains. The next bands at 1238 cm^−1^ and 1080 cm^−1^ are related with P=O asymmetrical and symmetrical stretching vibrations of PO_2_ phosphodiester groups from phosphorylated molecules. In the same way, the band at 1030 cm^−1^ is responsible for the C-O stretching vibration coupled with C-O bending of the C-OH groups of carbohydrates (including glucose, fructose, and glycogen). A band on the interval at 986–992 cm^−1^ is associated with the ribose phosphate main chain, whereas the band at 966 cm^−1^ arises from the stretching vibration of DNA backbone.

**Figure 4:**
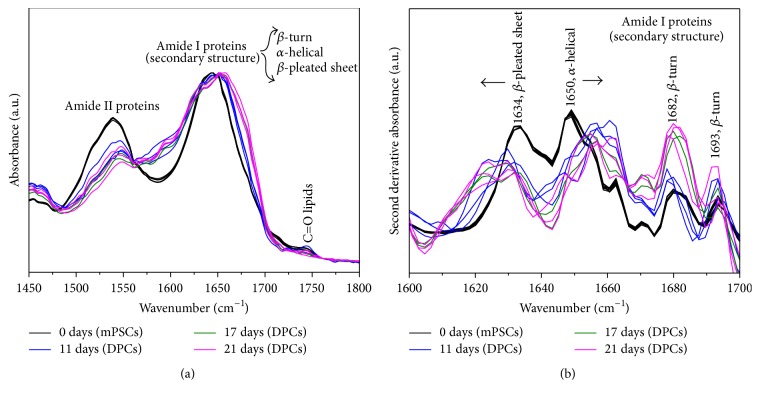
FTIR spectra of mouse pluripotent stem cells (mPSCs) and Differentiated Pancreatic Cells (DPCs) at different maturation stages (11, 17, and 21 days) depicted in the amide I and amide II region (*N* = 3). (a) Raw spectra. (b) Second derivative of absorbance.

Figure 4(a) shows the FTIR raw spectra of mPSCs and DPCs at different maturation stages depicted in the amide I and amide II region (1500–1700 cm^−1^). Broadening of the amide I band at 1650 cm^−1^ (C=O stretching vibration) of DPCs compared with the corresponding amide I band of mPSCs was observed. Such observed broadening of the amide I band of DPCs is also accompanied by a decrease in intensity of the amide II band of DPCs too. Thus, the intensity of the amide II band at around 1540 cm^−1^ in DPCs was significantly reduced compared with the corresponding mPSCs band.

Figure 4(b) shows the second derivative of the FTIR spectra of mPSCs and DPCs depicted in the amide I region (1600–1700 cm^−1^). The observed bands are related with components of the secondary structure of proteins, such as *β*-pleated-sheets (1634 cm^−1^), *α*-helices (1650 cm^−1^), and *β*-turns (1682 and 1693 cm^−1^), which are sensitive to structural and conformational changes. Particularly, an increment in the intensity of the *β*-turns and changes in the intensity and frequency of the *α*-helices of DPCs compared with mPSCs are observed.

**Figure 5:**
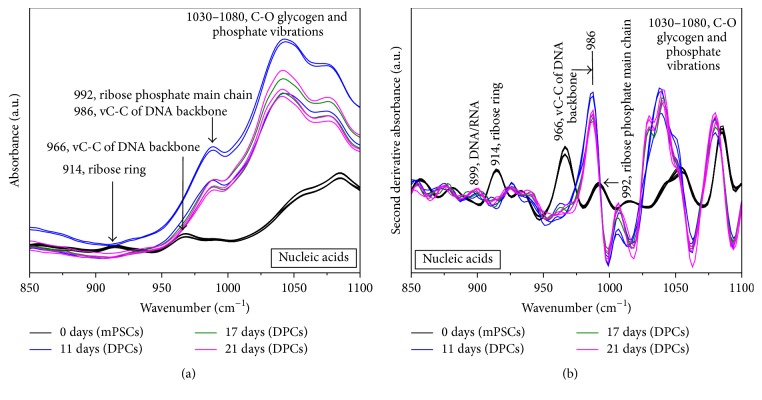
FTIR spectra of mouse pluripotent stem cells (mPSCs) and Differentiated Pancreatic Cells (DPCs) at different maturation stages (11, 17, and 21 days) depicted in the nucleic acids region (*N* = 3). (a) Raw spectra. (b) Second derivative of absorbance.

Figure 5(a) shows the raw FTIR spectra of mPSCs and DPCs depicted in the nucleic acids region (850–1100 cm^−1^). The band at 1080 cm^−1^ is associated with symmetrical stretching vibrations of PO2 phosphodiester groups, and another band at 1030 cm^−1^ could be associated with glycogen. Two overlapped bands at 992 cm^−1^ (ribose phosphate main chain) and 986 cm^−1^ (stretching vibration C-C of DNA backbone) were observed. Finally, we appreciated two weak bands at 966 cm^−1^ and 914 cm^−1^ associated with the stretching vibration C-C of DNA backbone and the vibration of ribose ring, respectively.

Figure 5(b) shows the second derivative of the FTIR spectra of mPSCs and DPCs in the nucleic acids region (850–1100 cm^−1^). We observed more definite bands compared with that observed in the raw spectra. Such as a significant increase in the intensity of the bands corresponding to glycogen and phosphate groups between 1030 cm^−1^ and 1080 cm^−1^ on DPCs compared to mPSCs. Likewise, we noted that the spectral signatures of the RNA and DNA content of mPSCs corresponding to ribose phosphate main chain mode (992 cm^−1^) and ribose ring mode (914 cm^−1^) decreased along mPSCs differentiation; on the other hand, the band corresponding to DNA C-C stretching of the backbone and RNA ribose phosphate main chain modes (966 cm^−1^) showed a shift toward 986 cm^−1^ and its intensity increased significantly on DPCs. In the same way, at the end of the differentiation process (day 21) a band at 899 cm^−1^ due to DNA/RNA was observed on DPCs.

## 4. Discussion

Because of the SCs ability to originate various specialized cells under specific culture conditions, the use of these cells has been proposed in the field of medical engineering and regenerative medicine. In this sense, it has been necessary to develop noninvasive and fast techniques to identify different SCs lineages as well as the maturation stages along their process of differentiation.

For this reason, in this work we differentiated mPSCs toward pancreatic cells showing the genetic and protein features at specific time points of differentiation employing RT-qPCR and immunocytochemistry; furthermore, we demonstrated the utility of FTIR spectroscopy to characterized the differentiation process of DPCs, recognizing this method as a rapid and noninvasive technique that requires a small quantity of sample, which could be very useful on the practice before implantation.

Once mPSCs differentiation toward DPCs was standardized, we proceeded to quantify the relative expression of each transcript (*Nanog, SOX2, SOX17, Pdx1, Ins-1, Ins-2, and Gcg*) at specific maturation stages (0, 11, 17, and 21 days of differentiation) (Figure 1).

In 2003 Chambers and Matsui reported the identification of* Nanog* as a new member of the ESCs stage. In this sense, it has been used to identify PSCs, due to the fact that* in vivo* its expression is firstly detected in the interior cells of compacted morula and in the ICM, and* in vitro* it marks all pluripotent cell lines (murine and human). It has also been reported that* Nanog* ablation in ESCs causes differentiation into endoderm lineages; therefore,* Nanog* is essential for maintaining the pluripotent status* in vivo* and* in vitro*, and its principal function is to prevent endoderm differentiation [20]. On the other hand, Takahashi and Yamanaka have stated that* SOX2* is part of the core regulatory network of transcription factors required for pluripotency maintenance and cellular reprogramming [21]. For this reason, in this research the expression of* Nanog* and* SOX2* in mPSCs confirmed the pluripotency status of the cultured SCs, as well as its differentiation when these cells missexpressed both genes at day 11; furthermore, the loss of* Nanog* expression is an important factor to guide the differentiation to endoderm germline.

In regard to the expression of endodermal germ line genes (*SOX17* and* Pdx1*), it is known that* SOX17* is a key regulator in the formation of definitive endoderm in many vertebrate species including the mouse, where it is expressed through gastrulation in both ventral and dorsal endoderm of the epiblast [22, 23]. In contrast to Chen et al. who reported that murine undifferentiated ESCs did not express* SOX17* [13], we could detect* SOX17* expression on mPSCs, probably due to the fact that recent researchers have found that* SOX17* is expressed within the ICM of the mouse blastocyst and within ESCs cultures, where it is a central component of the transcriptional network governing differentiation [24]. But nevertheless, the same authors also reported that the obtained pancreatic-like cells were* SOX17*+ at days 5, 6, and 16 of culture [13], and similarly we showed the expression of* SOX17* from day 0 to day 17.

*Pdx1* is one of the earliest known markers of the developing pancreas in both mice and humans [25], and it is also essential for the maintenance of the function of *β* cells. In this work we have shown that this gene was detectable until day 11 of differentiation, results that are comparable to those obtained by Phillips et al. who differentiated hESCs and showed that* Pdx1* expression is detected as early as day 12 of differentiation [26]. However, according to Gu et al.,* Pdx1* remains expressed during pancreas development and subsequently after birth; thereafter,* Pdx1* expression is restricted to mature *β* cells [27], and we observed that* Pdx1* expression decreased until it was undetectable at day 21, what make us think that the cells that we obtained are not mature cells.

About the expression of genes related to *β* cell (*Ins-1* and* Ins-2*), it has been reported that insulin genes in mice and rats form a two-gene system composed of preproinsulin 2 (*Ins2*), an ortholog to the insulin genes in other mammals, and preproinsulin 1 (*Ins1*), a rodent specific retrogene. Both genes are expressed in the pancreas and both encode proinsulin peptides [28]. Herein we demonstrated that at day 17 of differentiation, the expression of both insulins was evidenced; nevertheless at day 21 the expression considerably increased, results that are similar to those obtained by Shi et al. who induced the differentiation of mESC into pancreatic *β* cells and detected the expression of* Ins-1* at day 14 of differentiation [29]. Likewise, our results agree with Liu and Lee who differentiated mESC to pancreatic cells, detecting pancreatic *β* cell markers (*Ins-1* and* Ins-2*) at day 7 [30]. Although some differences are seen in the days of insulins expression, all works have reported the expression of both insulins at the final stages of differentiation; these changes probably result from the diverse differentiation protocols that have been used by different research groups.

Glucagon (*Gcg*) is the gene coding for glucagon-like peptide 1 (GLP-1) in the intestine but also encodes glucagon in *α* cells of the pancreatic islets [28]. As well as the expression of insulin, the expression of* Gcg* was evident at day 17 of differentiation, increasing thereafter at day 21, agreeing our results with Liu and Lee who detected the expression of* Gcg* at the end of the differentiation process [30]. In the same way, our results were similar to those obtained by Chen et al. who reported that the highest expression of* Ins-1*,* Ins-2,* and* Gcg* was observed after day 20 of differentiation [13]. Suggesting that the obtained cells before day 20 are still at a relatively early phase of differentiation.

Subsequent to the RT-qPCR assays, immunofluorescence staining was performed in mPSCs and DPCs (Figure 2). It has been reported that Oct4, SOX2, and Nanog are typical pluripotency markers [21] that retain the undifferentiation state in both early embryos and ESCs, reason by which in this work we look for Nanog and SOX2 expression on mPSCs (day 0 of differentiation), demonstrating that our mPSCs cultures expressed these proteins, which confirmed their pluripotency state. Thereafter, as expected, these pluripotency markers were undetectable on DPCs at day 21 of differentiation.

Concerning pancreas proteins expression, we confirmed that the obtained EBs were composed of DPCs producers of insulin and glucagon proteins, results that are quite similar to that reported by Chen et al. and Jiang et al., who differentiated mESCs and hESCs toward pancreatic cells, respectively, detecting the expression of both proteins at day 20 of differentiation [13, 31].

According to infrared vibrational spectroscopy, the measured FTIR spectrum of mPSCs was quite similar to the ESCs spectrum reported by Ami et al. [16]; thereafter marked changes in the intensities and frequencies of the absorbance bands between mPSCs and DPCs were observed, particularly in proteins and nucleic acid bands, reflecting structural differences between these cell lineages (Figure 3). Moreover, Ami et al. also reported these spectral changes during ESCs differentiation via EBs formation, confirming with the aforementioned the obtention of EBs from mPSCs by vibrational spectroscopy.

Concordant with some SCs differentiation studies, the extent and local detectability of the FTIR spectral variations clearly are consistent with the conformational change in proteins [10, 32]. Particularly, the FTIR absorption of the amide group at 1500 to 1700 cm^−1^ (Figure 4(a)) may indicate the expression of new proteins in DPCs and also gives information of the total cell protein content compared with mPSCs.

In addition to that, analyzing the second derivative of the amide I band from 1600 to 1700 cm^−1^ (Figure 4(b)), we can observe variations of the relative intensity of components associated with the secondary structure of proteins [33], as *β*-pleated-sheets, *α*-helices, and *β*-turns when differentiation took place, suggesting again that specific proteins were expressed and reordered by the DPCs along differentiation. Compared with those reported by Ami et al. [16], we saw an increase and shift of the *α*-helix band and an overexpression of *β*-turn structures that may suggest the characteristics of a pancreatic phenotype. Interestingly, according to Oyamada et al., this overexpression of *β*-turns is related with an increase of gap junctions during ES cell differentiation [34].

Likewise, we observed prominent changes in frequency and intensity of the bands at 1080 and 1238 cm^−1^ on DPCs compared with mPSCs that may demonstrate an altered absorption of the ring vibration of carbohydrates, which could be assigned to the sugar moieties of nucleic acids, to changed content of metabolic sugar molecules in the cells, such as glucose, or the absorbance of glycogen which start to increase during ESCs differentiation. Moreover, according to Walsh et al., the spectral interval from 1030 to 1080 cm^−1^, arises from glycogen and symmetric phosphate stretching vibrations, constituting a good indicator of alterations in the secondary structure of DNA, highlighting this region as pivotal in the differentiation process [35, 36].

Furthermore, prominent changes in the FTIR spectra in the nucleic acids region from 850 to 1100 cm^−1^ were noted (Figure 5(a)). These variations in intensity and frequency during differentiation process may also indicate changes in RNA content owing to upregulation or downregulation of genes along differentiation process or suggest that the transcriptional switch of the genome started along differentiation [33].

Second derivate analysis of nucleic acids on the region between 850 and 1100 cm^−1^ (Figure 5(b)) showed a significant increment of the intensity of the bands associated with glycogen and phosphate vibrations (1030–1080 cm^−1^) on DPCs compared to mPSCs, probably due to the fact that mESCs self-renewal is enhanced by partial inhibition of glycogen synthase kinase-3 (Gsk3), and when ESCs lose their state of undifferentiation, Gsk3 activates and increases glycogen levels in differentiated cells [37, 38].

With respect to the spectral signatures of the RNA and DNA content of mPSCs, we observed changes along the differentiation process, and similar to Ami et al. the bands corresponding to ribose ring mode (914 cm^−1^) and ribose phosphate main chain mode (992 cm^−1^) of mPSCs decreased during differentiation, possibly due to the fact that polyADP-ribose (pADPr) contributes to control stem cell self-renewal and oocyte localization by regulating DE-cadherin translation [39], so it is normal to find higher content of ribose on mPSCs.

In the same way, during differentiation process an increase and shift of the band 966 cm^−1^ toward 986 cm^−1^ was observed, possibly due to the emergence of a new band that might be assigned to RNA as reported Ami et al. and Banyay et al.; likewise, at the end of the differentiation protocol we appreciated the presence of a new component at 899 cm^−1^, which can be assigned to a vibrational mode of A-DNA, indicating that also a DNA/RNA hybrid started to be present [16, 40]. These spectral changes indicated that mRNA translation was taking place and these specific proteins were produced, reflecting the appearance of a new phenotype according to protein analysis.

Finally, this methodology could be translated into a high-throughput clinical setting by combining this kind of spectral results with computational analysis, to elucidate the state of cell differentiation [41–43]. From our point of view, FTIR spectroscopy will result in a powerful tool in the study and diagnostic of biological systems (as differentiated cells), once the combination with standard (gene/protein expression) and computational-chemometrical methods is possible.

## 5. Conclusion

In this study we obtained DPCs from mPSCs, which according to the genetic and phenotypic analysis passed through the chronological stages of embryonic pancreas development.

FTIR spectroscopy is a rapid, noninvasive, accurate, and efficient technique to analyze differentiated cells from mPSCs. In accordance with all the aforementioned, the infrared spectral changes provide a new biophysical parameter based on molecular markers indicating the differentiation process of mPSCs to specialized cells.

## Figures and Tables

**Table 1:** Nucleotide sequences of primer pairs used for real-time qPCR.

Gene	Forward 5′-3′	Reverse 5′-3′
*Nanog*	TCTCTCAGGCCCAGCTGTGT	CGCTTGCACTTCATCCTTTG
*SOX2*	AACCGATGCACCGCTACG	TTGACCACAGAGCCCATGG
*SOX17*	CTTCCCTACCAGGGACACGA	ACTGCTTCTGGCCCTCAGGT
*Pdx1*	GGTGCTTACACAGCGGAACC	CGGTCAAGTTCAACATCACTGC
*Insulin-1*	ACAGCATCTTTGTGGTCCCC	CAGCACTGATCCACAATGCC
*Insulin-2*	GCTCTTCCTCTGGGAGTCCC	AAGGTCTGAAGGTCACCTGCTC
*Glucagon*	GCCACTCACAGGGCACATT	GTCCCTTCAGCATGCCTCTC
*ppia*	CCAGGATTCATGTGCCAGG	GCCATCCAGCCATTCAGTCT

## References

[1] Sykova E., Forostyak S. (2013). Stem cells in regenerative medicine. *Laser Therapy*.

[2] Bajada S., Mazakova I., Richardson J. B., Ashammakhi N. (2008). Updates on stem cells and their applications in regenerative medicine. *Journal of Tissue Engineering and Regenerative Medicine*.

[3] Vázquez-Zapién G. J., Sánchez Monroy V., Chirino López Y. I., Mata-Miranda M. M. (2013). Morphological, genetic and protein characterization in the differentiation of embryonic stem cells to early pancreatic cells. *International Journal of Morphology*.

[4] Kroon E., Martinson L. A., Kadoya K. (2008). Pancreatic endoderm derived from human embryonic stem cells generates glucose-responsive insulin-secreting cells *in vivo*. *Nature Biotechnology*.

[5] Maeshima A., Nakasatomi M., Nojima Y. (2014). Regenerative medicine for the kidney: renotropic factors, renal stem/progenitor cells, and stem cell therapy. *BioMed Research International*.

[6] Vázquez-Zapién G. J., Rojas-López M., Delgado-Macuil R. J., Martínez-Nava L. R., Pérez-Ishiwara D. G., Mata-Miranda M. M. (2014). Histologic and spectroscopic study of pluripotent stem cells after implant in ocular traumatic injuries in a murine model. *Stem Cell Research and Therapy*.

[7] World Health Organization (2015). *Fact Sheet*.

[8] Márquez-Aguirre A. L., Canales-Aguirre A. A., Padilla-Camberos E., Esquivel-Solis H., Díaz-Martínez N. E. (2015). Development of the endocrine pancreas and novel strategies for *β*-cell mass restoration and diabetes therapy. *Brazilian Journal of Medical and Biological Research*.

[9] Rostovskaya M., Bredenkamp N., Smith A. (2015). Towards consistent generation of pancreatic lineage progenitors from human pluripotent stem cells. *Philosophical Transactions of the Royal Society B: Biological Sciences*.

[10] Downes A., Mouras R., Elfick A. (2010). Optical spectroscopy for noninvasive monitoring of stem cell differentiation. *Journal of Biomedicine and Biotechnology*.

[11] Ku H. T., Zhang N., Kubo A. (2004). Committing embryonic stem cells to early endocrine pancreas in vitro. *Stem Cells*.

[12] Kubo A., Shinozaki K., Shannon J. M. (2004). Development of definitive endoderm from embryonic stem cells in culture. *Development*.

[13] Chen C., Chai J., Singh L. (2011). Characterization of an in vitro differentiation assay for pancreatic-like cell development from murine embryonic stem cells: detailed gene expression analysis. *Assay and Drug Development Technologies*.

[14] Mantsch H. H., Yang P. W., Casal H. L. (1986). Infrared spectrometry of living systems: current trends and perspectives. *Journal of Molecular Structure*.

[15] Baker M. J., Trevisan J., Bassan P. (2014). Using Fourier transform IR spectroscopy to analyze biological materials. *Nature Protocols*.

[16] Ami D., Neri T., Natalello A. (2008). Embryonic stem cell differentiation studied by FT-IR spectroscopy. *Biochimica et Biophysica Acta (BBA)—Molecular Cell Research*.

[17] Chan J. W., Lieu D. K. (2009). Label-free biochemical characterization of stem cells using vibrational spectroscopy. *Journal of Biophotonics*.

[18] Pijanka J. K., Kumar D., Dale T. (2010). Vibrational spectroscopy differentiates between multipotent and pluripotent stem cells. *Analyst*.

[19] Heraud P., Ng E. S., Caine S. (2010). Fourier transform infrared microspectroscopy identifies early lineage commitment in differentiating human embryonic stem cells. *Stem Cell Research*.

[20] Carlson B. M. (2010). *Stem Cell Anthology*.

[21] Takahashi K., Yamanaka S. (2006). Induction of pluripotent stem cells from mouse embryonic and adult fibroblast cultures by defined factors. *Cell*.

[22] Spence J. R., Lange A. W., Lin S.-C. J. (2009). Sox17 regulates organ lineage segregation of ventral foregut progenitor cells. *Developmental Cell*.

[23] Moody S. A. (2015). *Principles of Developmental Genetics*.

[24] Niakan K. K., Ji H., Maehr R. (2010). Sox17 promotes differentiation in mouse embryonic stem cells by directly regulating extraembryonic gene expression and indirectly antagonizing self-renewal. *Genes & Development*.

[25] Kaneto H., Miyatsuka T., Kawamori D., Matsuoka T.-A. (2007). Pleiotropic roles of PDX-1 in the pancreas. *Review of Diabetic Studies*.

[26] Phillips B. W., Hentze H., Rust W. L. (2007). Directed differentiation of human embryonic stem cells into the pancreatic endocrine lineage. *Stem Cells and Development*.

[27] Gu G., Dubauskaite J., Melton D. A. (2002). Direct evidence for the pancreatic lineage: NGN^3+^ cells are islet progenitors and are distinct from duct progenitors. *Development*.

[28] Chiang Y.-T. A., Ip W., Jin T. (2012). The role of the Wnt signaling pathway in incretin hormone production and function. *Frontiers in Physiology*.

[29] Shi Y., Hou L., Tang F. (2005). Inducing embryonic stem cells to differentiate into pancreatic *β* cells by a novel three-step approach with activin A and all-trans retinoic acid. *Stem Cells*.

[30] Liu S. H., Lee L. T. (2012). Efficient differentiation of mouse embryonic stem cells into insulin-producing cells. *Experimental Diabetes Research*.

[31] Jiang W., Shi Y., Zhao D. (2007). In vitro derivation of functional insulin-producing cells from human embryonic stem cells. *Cell Research*.

[32] Cao J., Ng E. S., McNaughton D. (2013). The characterisation of pluripotent and multipotent stem cells using fourier transform infrared microspectroscopy. *International Journal of Molecular Sciences*.

[33] Chen Y.-J., Cheng Y.-D., Liu H.-Y., Lin P.-Y., Wang C.-S. (2006). Observation of biochemical imaging changes in human pancreatic cancer tissue using Fourier-transform infrared microspectroscopy. *Chang Gung Medical Journal*.

[34] Oyamada Y., Komatsu K., Kimura H., Mori M., Oyamada M. (1996). Differential regulation of gap junction protein (connexin) genes during cardiomyocytic differentiation of mouse embryonic stem cells in vitro. *Experimental Cell Research*.

[35] Walsh M. J., Fellous T. G., Hammiche A. (2008). Fourier transform infrared microspectroscopy identifies symmetric PO_2_
^−^ modifications as a marker of the putative stem cell region of human intestinal crypts. *Stem Cells*.

[36] Walsh M. J., Hammiche A., Fellous T. G. (2009). Tracking the cell hierarchy in the human intestine using biochemical signatures derived by mid-infrared microspectroscopy. *Stem Cell Research*.

[37] Wray J., Kalkan T., Gomez-Lopez S. (2011). Inhibition of glycogen synthase kinase-3 alleviates Tcf3 repression of the pluripotency network and increases embryonic stem cell resistance to differentiation. *Nature Cell Biology*.

[38] Gazi E., Dwyer J., Gardner P. (2003). Applications of Fourier transform infrared microspectroscopy in studies of benign prostate and prostate cancer. A Pilot Study. *Journal of Pathology*.

[39] Ji Y., Tulin A. V. (2012). Poly (ADP-ribose) controls DE-cadherin-dependent stem cell maintenance and oocyte localization. *Nature Communications*.

[40] Banyay M., Sarkar M., Gräslund A. (2003). A library of IR bands of nucleic acids in solution. *Biophysical Chemistry*.

[41] Trevisan J., Angelov P. P., Scott A. D., Carmichael P. L., Martin F. L. (2013). IRootLab: a free and open-source MATLAB toolbox for vibrational biospectroscopy data analysis. *Bioinformatics*.

[42] Trevisan J., Angelov P. P., Carmichael P. L., Scott A. D., Martin F. L. (2012). Extracting biological information with computational analysis of Fourier-transform infrared (FTIR) biospectroscopy datasets: current practices to future perspectives. *Analyst*.

[43] Theophilou G., Paraskevaidi M., Lima K. M., Kyrgiou M., Martin-Hirsch P. L., Martin F. L. (2015). Extracting biomarkers of commitment to cancer development: potential role of vibrational spectroscopy in systems biology. *Expert Review of Molecular Diagnostics*.

